# Aboriginal and Torres Strait Islander Women’s Access to and Interest in mHealth: National Web-based Cross-sectional Survey

**DOI:** 10.2196/42660

**Published:** 2023-03-06

**Authors:** Sarah Jane Perkes, Billie Bonevski, Kerry Hall, Joerg Mattes, Catherine Chamberlain, Jessica Bennett, Robyn Whittaker, Kerrin Palazzi, David Lambkin, Michelle Kennedy

**Affiliations:** 1 College of Medicine and Public Health Flinders University Bedford Park Australia; 2 First Peoples Health Unit, Griffith University Southport Australia; 3 School of Medicine and Public Health Faculty of Health and Medicine University of Newcastle Newcastle Australia; 4 Hunter Medical Research Institute New Lambton Heights Australia; 5 Indigenous Health Equity Unit, Melbourne School of Population and Global Health The University of Melbourne Melbourne Australia; 6 National Institute for Health Innovation The University of Auckland Auckland New Zealand

**Keywords:** mHealth, Aboriginal, Torres Strait Islander, public health, health literacy, digital literacy, Australia, native, cross-sectional, national survey, technology use, technology ownership, digital device, mobile device, usage, adoption, acceptance, digital divide

## Abstract

**Background:**

Health programs delivered through digital devices such as mobile phones (mobile health [mHealth]) have become an increasingly important component of the health care tool kit. Aboriginal and Torres Strait Islander women of reproductive age are likely to be caring for children and family members and needing health care, but little is known about their access to and interest in mHealth.

**Objective:**

The objectives of this study were to investigate Aboriginal and Torres Strait Islander women’s ownership of digital devices, access to the internet, current mHealth use, and interest and preferences for future mHealth. We examined the factors (age, remoteness, caring for a child younger than 5 years, and level of education) associated with the ownership of digital devices, use of internet, and interest in using a mobile phone to improve health. This study also examines if women are more likely to use mHealth for topics that they are less confident to talk about face-to-face with a health professional.

**Methods:**

A national web-based cross-sectional survey targeting Aboriginal and Torres Strait Islander women of reproductive age (16-49 years) was performed. Descriptive statistics were reported, and logistic regressions were used to examine the associations.

**Results:**

In total, 379 women completed the survey; 89.2% (338/379) owned a smartphone, 53.5% (203/379) a laptop or home computer, 35.6% (135/379) a tablet, and 93.1% (353/379) had access to the internet at home. Most women used social media (337/379, 88.9%) or the internet (285/379, 75.2%) everyday. The most common modality used on the mobile phone for health was Google (232/379, 61.2%), followed by social media (195/379, 51.5%). The most preferred modality for future programs was SMS text messaging (211/379, 55.7%) and social media (195/379, 51.4%). The most preferred topics for future mHealth programs were healthy eating (210/379, 55.4%) and cultural engagement (205/379, 54.1%). Women who were younger had greater odds of owning a smartphone, and women with tertiary education were more likely to own a tablet or laptop. Older age was associated with interest to use telehealth, and higher educational attainment was associated with interest for videoconferencing. Most women (269/379, 70.9%) used an Aboriginal medical service and overall reported high rates of confidence to discuss health topics with a health professional. Overall, women showed a similar likelihood of selecting a topic in mHealth whether they were or were not confident to talk to a health professional about that.

**Conclusions:**

Our study found that Aboriginal and Torres Strait Islander women were avid users of the internet and had strong interest in mHealth. Future mHealth programs for these women should consider utilizing SMS text messaging and social media modalities and including content on nutrition and culture. A noteworthy limitation of this study was that participant recruitment was web-based (due to COVID-19 restrictions).

## Introduction

Aboriginal and Torres Strait Islander people experience inequitable health burden due to the continuing impacts of colonization, intergenerational trauma, and systemic racism experienced in Australia [1]. A number of health outcomes for Aboriginal and Torres Strait Islander people have remained steady or worsened over the past decade [2], including rates of mental illness [3], psychological distress [3], asthma [4], diabetes [5], cardiovascular disease [3], and chronic obstructive pulmonary disease [3], although gains have been made in other areas such as decreased smoking during pregnancy [6], antenatal visits [6,7], year 12 completion [8], and university attendance [8]. The life expectancy gap between Aboriginal and Torres Strait Islander women and non-Indigenous women is 7.6 years. Although the life expectancy of Aboriginal and Torres Strait Islander women has improved in recent decades, the gap still remains [9]. Addressing the social, cultural, and political determinants of health will lead to the greatest improvements in Aboriginal and Torres Strait Islander health outcomes [10]. A large-scale systemic reform that positions Aboriginal and Torres Strait Islander people as the decision makers for Aboriginal and Torres Strait Islander people is required [10].

Aboriginal and Torres Strait Islander women are “healers, storytellers, keepers of our kids, and truth-seekers” [11]. Aboriginal and Torres Strait Islander women have been instrumental in driving the change for Aboriginal and Torres Strait Islander people, including leading the mandate for Uluru Statement from the Heart [11]. Aboriginal and Torres Strait Islander women not only look after their own health but also the health of the collective: their community, grandchildren, parents, grandparents, children, and other family members [12]. The positive experiences of and role modeling by Aboriginal and Torres Strait Islander women to their children and others influence their development and behavior and often lead to better health outcomes for all of their community [13]. Health promotion programs targeting women’s health inevitably have important positive impacts on children and other community members.

There is strong evidence that health promotion programs developed by and for Aboriginal and Torres Strait Islander women are the most successful [10,14,15]. Aboriginal and Torres Strait Islander women can seek health care from Aboriginal Community Controlled Health Organizations (ACCHOs) or a mainstream public health service. Specific services for Aboriginal and Torres Strait Islander women’s and children’s health exist in both ACCHOs and mainstream services, though ACCHOs tend to outperform mainstream services in health and well-being outcomes [16]. One such exemplar of a women’s and children’s health promotion program developed by an ACCHO is the Waminda Dead or Deadly program [17]. This program has been running for over 10 years and aims to enhance cultural connection and health and well-being through a range of activities, including cooking groups with local ingredients, exercise groups (prenatal and postnatal), yarning groups, and lifestyle medicine. This program was designed by and for local Aboriginal and Torres Strait Islander women and therefore operates in a flexible way to meet local women’s needs [16]. Locally developed programs achieve positive health outcomes for Aboriginal and Torres Strait Islander women [16]. To supplement these programs and to reach women who may not have access, alternative modes of delivery could be beneficial.

The potential of technology in promoting health and well-being in general is significant, with low cost and wide reach, high acceptability, and equitability if digital inclusion is considered carefully. Telehealth, health websites, social media campaigns, web-based patient portals, health apps, SMS text messaging programs, and wearable devices are becoming important daily tools for health care nationally and internationally. The COVID-19 experience has reinforced how important it is to have alternatives to face-to-face health care. Global evidence suggests mobile health (mHealth) to be effective and acceptable to populations underserved by traditional primary health and public health campaigns [18,19]. mHealth may be particularly important for Aboriginal and Torres Strait Islander communities, given the high rate of mobile phone use [20,21] and barriers to accessing mainstream primary health care [22]. An important first step to developing and delivering effective mHealth interventions is gathering information about the population, including context, digital access, and interest in mHealth [23]. This information is critical to designing interventions that have sustained engagement [23], which many mHealth solutions fall short of [24]. To date, there is little information on the access, interest, and preferences in mHealth among Aboriginal and Torres Strait Islander women.

The aims of this study were as follows:

1. To describe Aboriginal and Torres Strait Islander women’s ownership of digital devices and access to the internet.
2. To describe Aboriginal and Torres Strait Islander women’s current use, interest, and preferences for future mHealth programs.
3. To examine factors (age, remoteness, caring for a child younger than 5 years, and level of education) associated with Aboriginal and Torres Strait Islander women’s access to digital devices and the internet and interest in using a mobile phone to improve health.
4. To examine if Aboriginal and Torres Strait Islander women are likely to be interested in using mHealth for health topics that they are not confident talking to a health professional about.

## Methods

### Study Design

A web-based cross-sectional survey design was selected, as we were interested in the experiences and views of a large sample of women at one point in time and to compare different variables at that point in time. We had planned to complete a portion of the surveys face-to-face; however, due to COVID-19 restrictions, web-based data collection was the most feasible approach. This study is reported according to the Checklist for Reporting Results of Internet E-Surveys [25].

### The Which Way? Study

This study is part of a larger study, the Which Way? study, a co-designed and co-owned research study with urban and regional Aboriginal and Torres Strait Islander communities [26-31]. The Which Way? study aims to improve care relating to smoking cessation by developing an Indigenous-led evidence base for smoking cessation to support Aboriginal and Torres Strait Islander women to be smoke-free during pregnancy and beyond. Detailed information on the larger study research prioritizations, governance, relationships, and methodologies can be found in the protocol paper [30].

### Study Participants

Aboriginal and Torres Strait Islander women of reproductive age (16-49 years) who were smokers or ex-smokers (any level of consumption) were invited to participate in this study. Smokers and ex-smokers were the eligibility criteria, as this study is a substudy of a large study on Aboriginal and Torres Strait Islander women’s preference for nonpharmacological approaches to smoking cessation.

### Procedures

Consent was obtained via a digital consent sheet using a tick box at the beginning of the survey. A copy of the Participant Information Sheet was provided via a hyperlink; progression through the survey was not granted until consent was provided. Participants were also informed of the approximate time required to complete the survey in the opening page of the survey. The survey was hosted on REDCap [32]. The database was accessible by authorized team members only. On completion of the survey, women were eligible to go in a draw for a chance to win an iPad. Women were recruited over a 3-month period between July 10, 2020 and October 10, 2020 inclusive. Participants were recruited via snowballing and targeted Facebook and Instagram paid advertising. The survey was promoted through social media by using both organic and paid advertisements. A Facebook page and an Instagram account were developed for the Which Way? study. The survey link was shared by the research team through professional contacts and by Aboriginal partner organizations via organizational social media pages and accounts. Paid advertising was used to increase reach on social media accounts. Advertising was specified for “location: Australia” and “Aboriginal peoples’ television network-Aboriginal title-smoking.” It was an open survey; all participants who accessed the link to the website were able to participate in the survey.

### Survey Instrument

#### Instrument Items

The survey included 36 items, of which 17 items are reported here. Branching logic was used to present questions that were relevant for each participant based on their previous responses. Generally, there was 1 survey item per page. The full survey took 10 minutes to complete. Women were required to complete each response to progress through the survey and were unable to return to their responses. Survey items and questions were developed in partnership with the partnering services. A draft survey was discussed among the research team, and partners then pretested with 15 Aboriginal and Torres Strait Islander women and community members known to the research team.

#### Participant Characteristics

The characteristics that were analyzed were (1) Aboriginal and Torres Strait Islander status, (2) age, (3) smoking status, (4) rurality (Accessibility and Remoteness Index of Australia), (5) use of Aboriginal Health Services, (6) education, (7) pregnancy status, (8) number of children living in the household, and (9) number of children younger than 5 years.

#### Access to Digital Devices and the Internet

1. Device ownership was determined by asking “What device(s) do you own?” (response options: iPad/tablet, laptop, or home computer; smartphone [iPhone or Android]; mobile phone [calls/text only]; I do not have access to any of these) [33].
2. Internet access was determined by asking “Where do you have internet access?” (response options: home, workplace, commuting/travel, community center, I don’t have internet access anywhere, somewhere else) [33].
3. Frequency of social media use was determined by asking “In the last 12 months, how often have you accessed social media?” (response options: not at all, about once a week, a few times per week, everyday) [34].
4. Frequency of internet use was determined by asking “In the last 12 months, how often have you accessed the internet for other things?” (response options: not at all, less than once a week, about once a week, a few times per week, everyday) [35].

#### Using Your Phone to Improve Health

1. Women were asked to indicate how they currently used their mobile phone for their health by responding to the following: “Do you currently use your mobile phone for anything to do with your health? (multiple selections allowed)” (response options: I use Google to find health information; I read posts or watch videos about health on social media; I use a health tracker; I use health apps; I use telehealth, eg, talk to a health worker on the phone for advice or treatment; I use text messages to communicate with health workers or have used a text messaging service for health; Other; No, I don’t use my phone for health) [34].
2. Women were asked to indicate what mHealth modalities they would be interested in using in the future by responding to “What type of mobile health would you like to use in the future if available? (multiple selections allowed)” (response options: text messaging service, eg, to help quit smoking or exercise or remind me to do something for my health; social media; health apps; health tracker; phone calls to talk to a health worker; videoconferencing to video call with a health worker; other; no, I wouldn’t use my phone for health in the future) [34].
3. Women were asked to indicate which 3 health topics they would be most interested in by responding to, “Pick 3 health topics that would be of most interest to you if using a mobile phone for your health” (response options: help me improve what I eat, engage with Aboriginal and Torres Strait Islander culture, show/teach me exercises, improve my mental health, help me to stop smoking, women’s health, help me limit or quit cannabis or other drugs, child’s health, help with family violence, help me limit drinking) [34].
4. To determine the participant’s confidence to talk to a health professional about different health topics in person, women were asked to respond to “Do you feel confident to talk with a doctor/health worker about the following health topics (women’s health, eating/diet, exercise, child’s health, mental health, quitting smoking, reducing alcohol, family violence, cannabis, or other drug use)?” (response options: yes, no, not relevant).

### Exclusion Criteria

Women were excluded from all analyses if they did not meet the inclusion criteria (ie, self-identifying as an Aboriginal or Torres Strait Islander woman, aged 16-49 years, and a current or ex-smoker) or if their survey was incomplete.

### Ethics Approval

Ethics approvals were granted by Aboriginal Health and Medical Research Council New South Wales (14541662), University of Newcastle (H-2020-0092), and the local health district ethics committee (2020/ETH02095).

### Analyses

The data were analyzed in SAS v9.4 (SAS Institute). Descriptive statistics are presented as count (%), mean (SD), and median (range). Age was categorized as <21 years, 21-34 years, and >34 years for descriptive statistics. Logistic regressions were used to examine the associations of age, remoteness, caring for a child younger than 5 years, and level of education with device ownership, mHealth modalities of interest, and mHealth topics of interest (top 3 selections only). A chi-squared test was used to examine the relationship between women’s interest in using mHealth for a health topic if they were not confident to discuss it in person with a health professional. An α level of .05 was specified for all tests and CIs.

Logistic regressions are presented as odds ratio (OR) (95% CI). For the logistic regressions, age was treated as continuous and ORs reported for increments of 5 years. Rurality was dichotomized into “major cities,” “regional,” and “remote” Australia. “Caring for a child younger than 5 years” was recoded as a binary response for the purposes of analysis. Level of education was collapsed into 3 categories and was presented as an overall Wald type-III *P* value and pairwise OR (95% CI) and *P* values for each level comparison with the reference (completed high school). “Education” was recoded into “did not complete high school,” “completed high school,” and “completed tertiary education” for the purpose of analysis. Complete case analysis was used for this study, given the relatively low number of participants being excluded due to missing data.

## Results

Data were collected for 865 women. Of these, 607 were eligible, 228 were excluded due to incomplete surveys, leaving 379 women included in the analyses.

### Participant Characteristics

Participant demographics are presented in Table 1. The mean age of the women was 31 years. More than half of the women lived in cities (194/379, 51.2%), 42.7% (162/379) in a regional area, and 6.1% (23/379) in a remote area. Most women used an Aboriginal health service (269/379, 70.9%).

**Table 1:** Demographic characteristics of Aboriginal and Torres Strait Islander women who were included in the survey (N=379).

Characteristics			Values
**Age (years)**			
	<21, n (%)	43 (11.3)	
	21-34, n (%)	196 (51.7)	
	>34, n (%)	140 (36.9)	
	Mean (SD)	31.0 (7.7)	
	Median (min, max)	32 (16, 49)	
**Remoteness, n (%)**			
	Major city	194 (51.2)	
	Regional	162 (42.7)	
	Remote	23 (6.1)	
**Level of education, n (%)**			
	Up to year 9	37 (9.8)	
	Year 10-11	102 (26.4)	
	Year 12	73 (19.3)	
	Current student at University/Technical and Further Education/apprentice	77 (20.3)	
	Trade certificate	40 (10.6)	
	University degree	50 (13.2)	
**Aboriginal and Torres Strait Island status, n (%)**			
	Aboriginal	357 (94.2)	
	Torres Strait Islander	7 (1.8)	
	Aboriginal and Torres Strait Islander	15 (3.9)	
**Use of Aboriginal Health Service, n (%)**			
	Yes	269 (70.9)	
	No	110 (29)	
**Children living in household, n (%)**			
	1-2	159 (42)	
	3 or more	129 (34)	
	None	91 (24)	
**Children living in household younger than 5 years, n (%)**			
	None	237 (63.5)	
	1 or more	142 (37.5)	

### Access to Digital Devices and the Internet

Approximately 89.2% (338/379) of the women owned a smartphone, 53.6% (203/379) a laptop, 35.6% (135/379) a tablet, and 16.4% (62/379) a mobile phone (calls and text only) (Table 2). Approximately 93.1% (353/379) of the women had access to the internet at home, and 88.9% (337/379) of the women used social media everyday.

**Table 2:** Device and internet access and frequency of social media and internet use (N=379).

Characteristics		Values, n (%)
**Device ownership**		
	Smartphone (iPhone or Android)	338 (89.2)
	Laptop or home computer	203 (53.6)
	iPad/tablet	135 (35.6)
	Mobile phone (calls/text only)	62 (16.4)
	I do not own any of these	2 (0.5)
**Access to the internet**		
	Home	353 (93.1)
	Workplace	165 (43.5)
	Commuting/travel	90 (23.7)
	Community center	48 (12.7)
	Somewhere else	17 (4.5)
	I don’t have access to the internet	8 (2.1)
**Use of social media in the last 12 months**		
	Everyday	337 (89)
	A few times per week	33 (8.7)
	Not at all	5 (1.3)
	About once a week	4 (1.1)
**Use of internet for other things in the last 12 months**		
	Everyday	285 (75.2)
	A few times per week	70 (18.5)
	About once a week	16 (4.2)
	Less than once a week	7 (1.8)
	Not at all	1 (0.3)

### Using the Phone to Improve Health

#### Current mHealth Modalities Used

The most common function used on the mobile phone for health was Google (232/379, 61.2%), followed by social media (182/379, 48%), health trackers (130/379, 34.3%), health apps (124/379, 32.7%), telehealth (116/379, 30.6%), and text messages (69/379, 18.2%) (Table 3).

**Table 3:** Mobile phone use and preferences for mobile health topics and functions (N=379).

Characteristics		Values, n (%)
**Current mobile health modalities used**		
	I use Google to find health information	232 (61.2)
	I read posts or watch videos about health on about health on social media	182 (48)
	I use a health tracker	130 (34.3)
	I use health apps	124 (32.7)
	I use telehealth	116 (30.6)
	I use text messages	69 (18.2)
	Other	8 (2.1)
	No, I do not use my phone for health	51 (13.5)
**Future mobile health modalities of interest**		
	A text messaging service	211 (55.7)
	Social media	195 (51.5)
	Health apps	184 (48.5)
	Health tracker	164 (43.3)
	Phone calls to talk to a health worker	152 (40.1)
	Videoconferencing to video call with a health worker	100 (26.4)
	Other	5 (1.3)
	No, I would not use my phone for health in the future	20 (5.3)
**Mobile health topics of interest**		
	Help me improve what I eat	210 (55.4)
	Engage with Aboriginal and Torres Strait Islander culture	205 (54.1)
	Show/teach me exercises	162 (42.7)
	Improve my mental health	155 (40.9)
	Help me to stop smoking	122 (32.2)
	Women’s health	61 (16.1)
	Help me limit or quit cannabis or other drugs	48 (12.7)
	Child’s health	45 (11.9)
	Help with family violence	28 (7.4)
	Help me limit drinking	23 (6.1)
	Other	9 (2.4)
	None of these topics interest me	5 (1.3)

#### Future mHealth Modalities of Interest

The most preferred mHealth modality for future health care was text messages (211/379, 55.7%), followed by social media (195/379, 51.5%), health apps (124/379, 48.5%), health trackers (164/379, 43.3%), telehealth (152/379, 40.1%), and videos (100/379, 26.4%) (Table 3).

#### mHealth Topics of Interest

The most preferred topic for future mHealth programs was healthy eating (210/379, 55.4%), followed by cultural engagement (205/379, 54.1%), exercise (162/379, 42.7%), mental health (155/379, 40.9%), stop smoking (122/379, 32.2%), women’s health (61/379, 16.1%), limit or quit cannabis or other drugs (48/379, 12.7%), child’s health (45/379, 11.9%), family violence (28/379, 7.4%), and limit unsafe drinking (23/379, 6.1%) (Table 3).

#### Confidence to Talk With a Health Professional About Different Health Topics

Women reported high rates of confidence to discuss all health topics with a health professional (Table 4).

**Table 4:** Confidence talking with a doctor/health worker about different health topics (N=379).

Health topics	Yes, n (%)	No, n (%)	Not relevant, n (%)
Women’s health	328 (86.6)	36 (9.5)	15 (4)
Eating/diet	314 (82.8)	51 (13.5)	14 (3.7)
Exercise	307 (81)	51 (13.5)	21 (5.5)
Child’s health	305 (80.5)	12 (3.2)	62 (16.4)
Mental health	285 (75.2)	50 (13)	44 (12)
Quitting smoking	265 (70)	49 (13.2)	65 (17.2)
Reducing alcohol	162 (42.7)	47 (12.4)	170 (44.9)
Family violence	135 (35.6)	79 (20.8)	165 (43.5)
Cannabis or other drug use	111 (29.2)	68 (17.9)	200 (52.7)

### Association Between Participant Characteristics and Device Ownership

For every 5-year increase in age, the odds of owning a smartphone decreased by 35% (OR 0.723, 95% CI 0.509-0.834; *P*<.001). Of those aged 16 to 21 years, 100% (43/43) owned a smartphone; of those aged 21-34 years, ownership was 90.8% (178/196); and of those aged 34-49 years, ownership was 83.6% (117/140). There was no association between owning a smartphone and level of education attainment or caring for a child younger than 5 years (Tables S1 and S2 in Multimedia Appendix 1).

Women who had completed tertiary education were almost twice as likely (OR 1.916, 95% CI 1.095-3.354; *P*=.02) to own an iPad or tablet compared to those whose highest education was high school completion. No other characteristics (age, remoteness, child younger than 5 years) were associated with ownership of an iPad or tablet (Tables S3 and S4 in Multimedia Appendix 1).

There was a statistically significant overall association with the level of education and ownership of a laptop or computer. Individuals who had completed tertiary education were more than twice as likely (OR 2.176, 95% CI 1.180-4.012; *P*=.01) to own a laptop or home computer compared to those who had completed high school. Those who had not completed high school were 69% less likely to own a laptop or computer compared to those who had completed high school (OR 0.310, 95% CI 0.190-0.506; *P*≤.001). No other characteristics (age, remoteness, or caring for a child younger than 5 years) were associated with ownership of a laptop or computer (Tables S5 and S6 in Multimedia Appendix 1).

The likelihood of owning a mobile phone (text and calls only) increased as age increased (per 5-year increase in age; OR 1.222, 95% CI 1.006-1.484; *P*=.04). No other characteristics (education, remoteness, or caring for a child younger than 5 years) were associated with ownership of an iPad or tablet (Tables S7 and S8 in Multimedia Appendix 1).

### Association Between Participant Characteristics and Future mHealth Modalities of Interest

For every 5-year increase in age, the odds of selecting telehealth as a future mHealth modality of interest increased by 22% (per 5-year increase in age; OR 1.232, 95% CI 1.065-1.425; *P*=.005). No other characteristics (education, remoteness, or caring for a child younger than 5 years) were associated with selecting telehealth as a preferred mHealth modality (Tables S9 and S10 in Multimedia Appendix 1). Women who had completed high school were more likely than those who did not complete high school to select videoconferencing as a future mHealth modality of interest (OR 0.497, 95% CI 0.284-0.872; *P*=.01). No other characteristics (tertiary education, remoteness, or caring for a child younger than 5 years) were associated with selecting videoconferencing as a preferred mHealth modality (Tables S11 and S12 in Multimedia Appendix 1). No statistically significant associations between participant characteristics and the selection of text messaging (Tables S13 and S14 in Multimedia Appendix 1), social media (Tables S15 and S16 in Multimedia Appendix 1), health apps (Tables S17 and S18 in Multimedia Appendix 1), or health tracker as preferred mHealth modalities (Tables S19 and S20 in in Multimedia Appendix 1) were found.

### Relationship Between Participant Characteristics and Preferred mHealth Topics

Women living in regional and remote areas were 43% less likely than those in urban areas to select cultural engagement as a topic (OR 0.437, 95% CI 0.287-0.666; *P*≤.001) (Tables S21 and S22 in Multimedia Appendix 1). No statistically significant associations were found between participant characteristics and the selection of healthy eating (Tables S23 and S24 in Multimedia Appendix 1) or exercise (Tables S25 and S26 in Multimedia Appendix 1).

### Relationship Between Confidence to Discuss a Health Topic With a Health Professional and Selecting That Topic for Future mHealth Interventions

Overall, women showed a similar likelihood of selecting a topic in mHealth whether they were or were not confident to talk to a health professional about that topic (Table S27 in Multimedia Appendix 1). Topics that showed a similar likelihood of being selected included diet, exercise, family violence, quitting smoking, cannabis and other drug use, mental health, women’s health, and children’s health. Reducing alcohol was the only topic that showed significance (*P*=.002). Notably, for most women (170/379, 44.9%), reducing alcohol was not a relevant health topic, and 42.7% (162/379) of the women were confident to discuss with a health provider. The number of women not confident to discuss reducing alcohol with a health provider was low (47/379, 12.4%) as such, and the number of women selecting alcohol reduction as an mHealth topic who were not confident to discuss with a health professional was also low (11/379, 2.9%).

## Discussion

### Principal Results

The findings of our study suggest that Aboriginal and Torres Strait Islander women have high access to smartphones and social media and their interest in using technology for health care is high. SMS text messaging was the most preferred mHealth modality.

### Strengths and Limitations

A strength of this study is that it was led and governed by Aboriginal researchers. MK, the senior author and lead investigator of the larger study, is a Wiradjuri woman working with a team of Aboriginal and Torres Strait Islander researchers. Aboriginal Health Services are full partners and co-owners of this research. The full details of the governance are available in the protocol for the larger study [30]. As a non-Indigenous researcher (SJP) leading the mHealth portion of the survey, it was important to be guided by Aboriginal leadership and partnership to ensure cultural safety and best practice of ethical standards of research with Aboriginal and Torres Strait Islander people [36,37].

A key limitation of this study is that all recruitments were conducted online and it is therefore biased toward people who have access to digital devices and the internet. We planned to complete a portion of the surveys in person; however, due to COVID-19 restrictions, this was not possible. Unsurprisingly, due to the recruitment strategy, access to devices and the internet was much higher in this study than in other available data. In this study, 99.5% (377/398) of the women owned either a smartphone or a mobile phone. It is unlikely that this proportion reflects all Aboriginal and Torres Strait Islander people, particularly people living in remote locations or in poverty. Two recent studies [38,39] with Aboriginal and Torres Strait Islander people reported lower access to mobile phones. In 1 study, 12.9% (39/301) of the women did not have access to a phone [38], and the other study reported frequent sharing of phones (rather than individual ownership), as is common practice in low-resource settings [39]. In this study, 93.1% (353/379) of the women had access to the internet at home compared to 72% of the Aboriginal and Torres Strait Islander people reported in the 2016 census [40]. Further, as this survey was embedded in a larger study on nonpharmacological smoking support, only current or former smokers were included, possibly creating further bias. Lastly, the usage of complete case analysis further limits the generalizability of this study to people with similar characteristics. Although these weaknesses may limit generalization, overall, these results offer useful insights into the type of mHealth modalities and topics of interest for the future development of mHealth programs.

### Comparison With Prior Work

In this study, SMS text messaging was found to be the most desired modality, with 55.7% (211/379) of the women reporting an interest in using SMS text messaging for future health care. Interestingly, SMS text messaging was the least currently utilized mHealth modality for health care at 18.2% (69/379). There were no significant associations found between participant characteristics and women selecting SMS text messaging as the desired modality; as such, SMS text messaging appears to be equally desirable by women of different ages (16-49 years), women living in cities and in regional or remote areas, women with and without young children, and women with different levels of educational attainment. Two studies using SMS text messaging with Aboriginal and Torres Strait Islander women in remote settings found high acceptability for SMS text messages but no difference in the clinical outcomes, including attendance for appointments for otitis media [41] and postpartum screening following gestational diabetes [39]. In a randomized controlled trial with Aboriginal and Torres Strait Islander families in urban and remote settings, SMS text messages were used to retain women; over 96.7% (180/186) of the children remained in the randomized controlled trial until their clinical end point at day 21 [38]. There is great potential for more effectively using SMS text messaging to reach Aboriginal and Torres Strait Islander women to improve health outcomes.

Social media was the second most preferred modality, with 51.5% (195/379) of the women selecting social media as an mHealth modality of interest for the future. Daily social media use among the women was high at 88.9% (337/379)—much higher than that reported in the rest of the population. A web-based survey in 2021 with 2000 Australians reported that 55% of the population used social media daily [42]—similar to the trend in a 2014 survey with 400 Aboriginal and Torres Strait Islander people, wherein 69% used Facebook compared with 40% of the other Australians [20]. The “Social Media Mob: Being Indigenous Online” report suggests that social media uptake is higher among Aboriginal and Torres Strait Islanders than the rest of the nation, including in remote and very remote areas [21]. Carlson and Frazer [21] outlined that Aboriginal and Torres Strait Islander people use social media for a range of positive reasons—to connect with friends and family, share jokes, seek love, find information, seek help, and political activism—but that ensuring psychological and cultural safety should be a priority.

Several Aboriginal and Torres Strait Islander–led social marketing campaigns for health promotion, such as Tackling Indigenous Smoking and Deadly Choices, create posts that appeal to positive emotions, photos and (short) videos, simple content, and real-time support, among other strategies [43]. In an ethnographic study of Deadly Choices, the 5 important principles for the success of the campaign were outlined: (1) create a dialogue, (2) build communities online and offline, (3) incentivize healthy web-based engagement, (4) celebrate Indigenous identity and culture, and (5) prioritize partnerships [44]. Future health initiatives on social media for Aboriginal and Torres Strait Islander women should lean on these findings.

The most preferred topics for future mHealth programs were healthy eating (210/379, 55.4%), followed by cultural engagement (205/379, 54.1%), exercise (162/379, 42.7%), and mental health (155/379, 40.9%). A recent qualitative study examined the types of health content that were shared among Aboriginal and Torres Strait Islander people through social media networks as well as how people engage with and are influenced by it [45]. They found that posts on mental health and nutrition were more commonly shared than posts on health topics where there is concern about stigma and shame, such as smoking and alcohol consumption [45]. The findings in our survey somewhat reflect those findings. Most of the topics associated with shame and stigma, including limiting or quitting cannabis or other drugs, family violence, and reducing alcohol, were in the bottom 4 (out of 9) health topics of interest. Although quitting smoking was the fifth (out of 9) popular choice, the difference may be due to most of the 20 participants in the qualitative study being smokers [45] compared to 37.5% (141/379) of the participants in our study being nonsmokers.

For topics that have concern for shame and stigma, it is suggested that negative messages that have been successful at a whole population level, such as quitting smoking, may need to be adapted for Aboriginal and Torres strait Islander mHealth initiatives [45]. The importance of centering positive cultural identity and narratives in mHealth initiatives is evident [43-45]. This was highlighted in a recent qualitative study that found Facebook posts celebrating culture and cultural achievements as well as challenging racism were mostly posted by women [45]. The popularity of embedding culture in mHealth programs was shown in the results of that study [45], with “culture” as the second most popular topic. The existing evidence suggests that future mHealth programs must integrate culturally relevant content. Further research to determine its effect on engagement and health outcomes is warranted.

Although smartphone ownership was relatively high at 89.2% (338/379) (similar to national figures of 92% [42]), ownership of other digital devices, including laptops, was low at 53.6% (203/379) (national figure is 78% [42]). Access to devices (as well as the internet and data), alongside affordability and digital ability are combined to provide a digital inclusion score (out of 100) [46]. The digital inclusion gap between Aboriginal and Torres Strait Islander people and the rest of the nation is 7.9 (55.1 compared to 63) [46]. Aboriginal and Torres Strait Islander people are less likely to have access to consistent, fast, and large amounts of data, more likely to be mobile-only users, and more likely to use prepaid data; these factors all decrease digital inclusion [46]. Digital inclusion facilitates efficient delivery and uptake of critical services, including health care, as well as employment and education opportunities [46]. Digital inclusion remains in The National Agreement on Closing the Gap as part of the Access to Information target (Target 17)—by 2026, Aboriginal and Torres Strait Islander people will have equal levels of digital inclusion. It is imperative that we seek to advance mHealth solutions developed for and by Aboriginal and Torres Strait Islander people.

### Conclusions

Aboriginal and Torres Strait Islander women are avid users of technology and have a strong interest in mHealth. New mHealth initiatives should consider having strong partnerships with ACCHOs and be designed by and for Aboriginal and Torres Strait Islander women to meet their digital and health needs. Nutrition and culture should be considered as topics of particular interest. Social media and SMS text messaging may be the most currently accessible and preferable modalities.

## Appendix

Multimedia Appendix 1 Supplementary tables.

## Abbreviations

ACCHOs: Aboriginal Community Controlled Health Organizations
mHealth: mobile health
OR: odds ratio

## Data Availability

The data sets generated during and analyzed during this study are not publicly available in line with Aboriginal and Torres Strait Islander health research ethical guidelines but are available from the corresponding author on reasonable request.

## References

[1] (2017). My Life My Lead - opportunities for strengthening approaches to the social determinants and cultural determinants of Indigenous health: report on the national consultations. Commonwealth of Australia, Department of Health.

[2] Mohamed J (2021). How can the new closing the gap dashboard highlight what indicators and targets are on track?. The Conversation.

[3] (2019). National Aboriginal and Torres Strait Islander health survey. Australian Bureau of Statistics.

[4] Brock C, McGuane J (2018). Determinants of asthma in Indigenous Australians: insights from epidemiology. Australian Indigenous Health Bulletin.

[5] Ride K, Burrow S (2022). Review of diabetes among Aboriginal and Torres Strait Islander people. Australian Indigenous HealthInfoNet.

[6] Taylor EL (2011). Aboriginal and Torres Strait Islander Mothers and Babies. NSW Public Health Bull.

[7] Kildea S, Gao Y, Hickey S, Nelson C, Kruske S, Carson A, Currie J, Reynolds M, Wilson K, Watego K, Costello J, Roe Y (2021). Effect of a Birthing on Country service redesign on maternal and neonatal health outcomes for First Nations Australians: a prospective, non-randomised, interventional trial. The Lancet Global Health.

[8] (2022). Australia: Aboriginal and Torres Strait Islander population summary. Australian Bureau of Statistics.

[9] (2018). Life tables for Aboriginal and Torres Strait Islander Australians. Australian Bureau of Statistics.

[10] (2022). Inquiry into the application of the United Nations declaration on the rights of Indigenous peoples in Australia. The Lowitja Institute.

[11] Reid T (2022). The power of the First Nations Matriarchy Warrior Women reckoning with the colony. Griffith Review.

[12] Burns J, Maling CN, Thomson N (2010). Summary of Indigenous female health. Australian Indigenous HealthInfoNet.

[13] (2018). Aboriginal and Torres Strait Islander women celebrated. Australian Bureau of Statistics.

[14] Pearson O, Schwartzkopff K, Dawson A, Hagger C, Karagi A, Davy C, Brown A, Braunack-Mayer A, Leadership Group guiding the Centre for Research Excellence in Aboriginal Chronic Disease Knowledge TranslationExchange (CREATE) (2020). Aboriginal community controlled health organisations address health equity through action on the social determinants of health of Aboriginal and Torres Strait Islander peoples in Australia. BMC Public Health.

[15] (2017). Aboriginal and Torres Strait Islander health organisations: online services report - key results 2015-16. Australian Institute of Health and Welfare.

[16] Fredericks B, Daniels C, Judd J, Bainbridge R, Clapham K, Longbottom M (2018). Gendered Indigenous health and well-being within the Australian health system: A review of the literature. UWA Research Repository.

[17] Fredericks B, Longbottom M, McPhail-Bell K, Worner F, Board W (2018). "Dead or deadly makes me feel healthy and fit": Findings from an Aboriginal women's health and wellbeing program within the shoalhaven region of New South Wales, Australia. Journal of Australian Indigenous Issues.

[18] Anderson-Lewis C, Darville G, Mercado RE, Howell S, Di Maggio S (2018). mHealth Technology Use and Implications in Historically Underserved and Minority Populations in the United States: Systematic Literature Review. JMIR Mhealth Uhealth.

[19] Dobson R, Whittaker R, Bartley H, Connor A, Chen R, Ross M, McCool J (2017). Development of a Culturally Tailored Text Message Maternal Health Program: TextMATCH. JMIR Mhealth Uhealth.

[20] Media usage amongst Aboriginal and Torres Strait Islander people (infographic). McNair Ingenuity Research.

[21] Carlson B, Frazer R (2018). Social media mob: being Indigenous online. Macquarie University.

[22] Davy C, Cass A, Brady J, DeVries J, Fewquandie B, Ingram S, Mentha R, Simon P, Rickards B, Togni S, Liu H, Peiris D, Askew D, Kite E, Sivak L, Hackett M, Lavoie J, Brown A (2016). Facilitating engagement through strong relationships between primary healthcare and Aboriginal and Torres Strait Islander peoples. Aust N Z J Public Health.

[23] (2019). Be He@lthy, Be Mobile Personas toolkit. World Health Organization International Telecommunication Union.

[24] Michie S, Yardley L, West R, Patrick K, Greaves F (2017). Developing and Evaluating Digital Interventions to Promote Behavior Change in Health and Health Care: Recommendations Resulting From an International Workshop. J Med Internet Res.

[25] Eysenbach G (2004). Improving the quality of Web surveys: the Checklist for Reporting Results of Internet E-Surveys (CHERRIES). J Med Internet Res.

[26] Kennedy M, Barrett E, Heris C, Mersha A, Chamberlain C, Hussein P, Longbottom H, Bacon S, Maddox R (2022). Smoking and quitting characteristics of Aboriginal and Torres Strait Islander women of reproductive age: findings from the Which Way? study. Med J Aust.

[27] Kennedy M, Heris C, Barrett E, Bennett J, Maidment S, Chamberlain C, Hussein P, Longbottom H, Bacon S, Field BG, Field B, Ralph F, Maddox R (2022). Smoking cessation support strategies for Aboriginal and Torres Strait Islander women of reproductive age: findings from the Which Way? study. Med J Aust.

[28] Kennedy M, Maddox R (2022). Miilwarranha (opening): introducing the Which Way? study. Med J Aust.

[29] Kennedy M, Maddox R (2022). Ngaaminya (find, be able to see): summary of key findings from the Which Way? project. Med J Aust.

[30] Bovill M, Chamberlain C, Bennett J, Longbottom H, Bacon S, Field B, Hussein P, Berwick R, Gould G, O'Mara Peter (2021). Building an Indigenous-Led Evidence Base for Smoking Cessation Care among Aboriginal and Torres Strait Islander Women during Pregnancy and Beyond: Research Protocol for the Which Way? Project. Int J Environ Res Public Health.

[31] Moltu C, Stefansen J, Svisdahl M, Veseth M (2012). Negotiating the coresearcher mandate - service users' experiences of doing collaborative research on mental health. Disabil Rehabil.

[32] Harris PA, Taylor R, Thielke R, Payne J, Gonzalez N, Conde JG (2009). Research electronic data capture (REDCap)--a metadata-driven methodology and workflow process for providing translational research informatics support. J Biomed Inform.

[33] Du JT, Haines J (2017). Indigenous Australians' information behaviour and internet use in everyday life: an exploratory study. Information Research.

[34] Krebs P, Duncan DT (2015). Health App Use Among US Mobile Phone Owners: A National Survey. JMIR Mhealth Uhealth.

[35] McCrabb S, Twyman L, Palazzi K, Guillaumier A, Paul C, Bonevski B (2019). A cross sectional survey of internet use among a highly socially disadvantaged population of tobacco smokers. Addict Sci Clin Pract.

[36] (2018). Keeping research on track II. National Health and Medical Research Council.

[37] (2018). Ethical conduct in research with Aboriginal and Torres Strait Islander peoples and communities: Guidelines for researchers and stakeholders. National Health and Medical Research Council.

[38] McCallum GB, Versteegh LA, Morris PS, Mckay CC, Jacobsen NJ, White AV, D'Antoine Heather A, Chang AB (2014). Mobile phones support adherence and retention of indigenous participants in a randomised controlled trial: strategies and lessons learnt. BMC Public Health.

[39] Kirkham R, MacKay D, Barzi F, Whitbread C, Kirkwood M, Graham S, Van Dokkum P, McIntyre HD, Shaw JE, Brown A, O'Dea K, Connors C, Oats J, Zimmet P, Boyle J, Maple-Brown Louise (2019). Improving postpartum screening after diabetes in pregnancy: Results of a pilot study in remote Australia. Aust N Z J Obstet Gynaecol.

[40] (2016). 2071.0 - Census of population and housing: reflecting Australia - stories from the census, 2016. Australian Bureau of Statistics.

[41] Phillips JH, Wigger C, Beissbarth J, McCallum GB, Leach A, Morris PS (2014). Can mobile phone multimedia messages and text messages improve clinic attendance for Aboriginal children with chronic otitis media? A randomised controlled trial. J Paediatr Child Health.

[42] (2021). Digital consumer trends. Deloitte.

[43] (2020). Social media and social networking. National Best Practice Unit: Tackling Indigenous Smoking.

[44] McPhail-Bell K, Appo N, Haymes A, Bond C, Brough M, Fredericks B (2018). Deadly Choices empowering Indigenous Australians through social networking sites. Health Promot Int.

[45] Hefler M, Kerrigan V, Henryks J, Freeman B, Thomas D (2019). Social media and health information sharing among Australian Indigenous people. Health Promot Int.

[46] (2021). Indigenous digital inclusion plan: discussion paper. Commonwealth of Australia: National Indigenous Australians Agency.

