# Supplementary material for: Aboriginal and Torres Strait Islander Women’s Access to and Interest in mHealth: National Web-based Cross-sectional Survey
Source: J Med Internet Res. 2023 Mar 6;25:e42660. doi: 10.2196/42660 (PMC10028504; doi:10.2196/42660)
Supplement: Multimedia Appendix 1 [file jmir_v25i1e42660_app1.docx]

**Supplementary tables**

**Table S1: Participant Characteristics for Owning a “Smart Phone”**

| *Characteristic* *Class/Statistic* *Unchecked* | | | *Checked* |
| --- | --- | --- | --- |
| *(n=41)* | | | *(n=338)* |
| Age | <21 |  | 43 (100.0%) |
|  | 21-34 | 18 (9.2%) | 178 (91%) |
|  | >34 | 23 (16%) | 117 (84%) |
| ARIA+ | Major Cities of Australia | 15 (7.7%) | 179 (92%) |
|  | Regional and Remote Australia | 26 (14%) | 159 (86%) |
| Do you have children under 5? | No | 29 (12%) | 208 (88%) |
|  | Yes | 12 (8.5%) | 130 (92%) |
| What education level have you completed? | Did Not Complete High School | 20 (14%) | 119 (86%) |
|  | Completed High School | 12 (8.0%) | 138 (92%) |
|  | Completed Tertiary Education | 9 (10%) | 81 (90%) |

**Table S2: Associations Between Characteristics and Owning a “Smart Phone”**

| *Characteristic* | *Crude* *(n=379)*  *OR* *(95%CI)* | *p* | *Adjusted* *(n=379)*  *OR* *(95%CI)* | *p* |
| --- | --- | --- | --- | --- |
| Age (+5 year increment) | 0.649 (0.512, 0.823) | 0.0003 | 0.651 (0.509, 0.834) | 0.0007 |
| ARIA+ (Regional and Remote Australia vs. Major Cities of Australia) | 0.512 (0.262, 1.002) | 0.0507 | 0.512 (0.255, 1.026) | 0.0592 |
| Do you have any children under 5? (Yes vs. No) | 1.510 (0.744, 3.063) | 0.2536 | 1.271 (0.607, 2.661) | 0.5247 |
| What education level have you completed? (Overall comparison*) |  | 0.2158* |  | 0.4526* |
| Competed Tertiary Education vs. Completed High School | 0.783 (0.316, 1.938) | 0.5962 | 1.124 (0.431, 2.930) | 0.8108 |
| Did Not Complete High School vs. Completed High School | 0.517 (0.243, 1.102) | 0.0878 | 0.685 (0.311, 1.509) | 0.3483 |

**Table S3: Participant Characteristics for Owning an “iPad / Tablet”**

| *Characteristic* *Class/Statistic* *Unchecked* | | | *Checked* |
| --- | --- | --- | --- |
| *(n=244)* | | | *(n=135)* |
| Age | <21 | 33 (77%) | 10 (23%) |
|  | 21-34 | 131 (67%) | 65 (33%) |
|  | >34 | 80 (57%) | 60 (43%) |
| ARIA+ | Major Cities of Australia | 124 (64%) | 70 (36%) |
|  | Regional and Remote Australia | 120 (65%) | 65 (35%) |
| Do you have children under 5? | No | 152 (64%) | 85 (36%) |
|  | Yes | 92 (65%) | 50 (35%) |
| What education level have you completed? | Did Not Complete High School | 102 (73%) | 37 (27%) |
|  | Completed High School | 99 (66%) | 51 (34%) |
|  | Completed Tertiary Education | 43 (48%) | 47 (52%) |

**Table S4: Associations Between Characteristics and Owning an “iPad / Tablet”**

| *Characteristic* | *Crude* *(n=379)*  *OR* *(95%CI)* | *p* | *Adjusted* *(n=379)*  *OR* *(95%CI)* | *p* |
| --- | --- | --- | --- | --- |
| Age (+5 year increment) | 1.165 (1.013, 1.340) | 0.0325 | 1.130 (0.973, 1.311) | 0.1097 |
| ARIA+ (Regional and Remote Australia vs. Major Cities of Australia) | 0.960 (0.630, 1.461) | 0.8474 | 1.105 (0.713, 1.712) | 0.6550 |
| Do you have any children under 5? (Yes vs. No) | 0.972 (0.629, 1.501) | 0.8978 | 1.056 (0.674, 1.655) | 0.8116 |
| What education level have you completed? (Overall comparison*) |  | 0.0005* |  | 0.0012* |
| Competed Tertiary Education vs. Completed High School | 2.122 (1.244, 3.619) | 0.0058 | 1.916 (1.095, 3.354) | 0.0228 |
| Did Not Complete High School vs. Completed High School | 0.704 (0.425, 1.168) | 0.1741 | 0.658 (0.393, 1.101) | 0.1113 |

**Table S5: Participant Characteristics for Owning a “Laptop / Home Computer”**

| *Characteristic* *Class/Statistic* *Unchecked* | | | *Checked* |
| --- | --- | --- | --- |
| *(n=176)* | | | *(n=203)* |
| Age | <21 | 18 (42%) | 25 (58%) |
|  | 21-34 | 94 (48%) | 102 (52%) |
|  | >34 | 64 (46%) | 76 (54%) |
| ARIA+ | Major Cities of Australia | 84 (43%) | 110 (57%) |
|  | Regional and Remote Australia | 92 (50%) | 93 (50%) |
| Do you have children under 5? | No | 105 (44%) | 132 (56%) |
|  | Yes | 71 (50%) | 71 (50%) |
| What education level have you completed? | Did Not Complete High School | 95 (68%) | 44 (32%) |
|  | Completed High School | 60 (40%) | 90 (60%) |
|  | Completed Tertiary Education | 21 (23%) | 69 (77%) |

**Table S6: Associations Between Characteristics and Owning a “Laptop / Home Computer***”*

| *Characteristic* | *Crude* *(n=379)*  *OR* *(95%CI)* | *p* | *Adjusted* *(n=379)*  *OR* *(95%CI)* | *p* |
| --- | --- | --- | --- | --- |
| Age (+5 year increment) | 1.015 (0.890, 1.158) | 0.8249 | 0.976 (0.843, 1.130) | 0.7474 |
| ARIA+ (Regional and Remote Australia vs. Major Cities of Australia) | 0.772 (0.515, 1.157) | 0.2099 | 0.929 (0.600, 1.439) | 0.7424 |
| Do you have any children under 5? (Yes vs. No) | 0.795 (0.524, 1.207) | 0.2821 | 0.803 (0.513, 1.258) | 0.3385 |
| What education level have you completed? (Overall comparison*) |  | <.0001* |  | <.0001* |
| Competed Tertiary Education vs. Completed High School | 2.190 (1.217, 3.942) | 0.0089 | 2.176 (1.180, 4.012) | 0.0127 |
| Did Not Complete High School vs. Completed High School | 0.309 (0.190, 0.501) | <.0001 | 0.310 (0.190, 0.506) | <.0001 |

**Table S7: Participant Characteristics for Owning a “Mobile Phone”**

| *Characteristic* *Class/Statistic* *Unchecked* | | | *Checked* |
| --- | --- | --- | --- |
| *(n=317)* | | | *(n=62)* |
| Age | <21 | 40 (93%) | 3 (7.0%) |
|  | 21-34 | 167 (85%) | 29 (15%) |
|  | >34 | 110 (79%) | 30 (21%) |
| ARIA+ | Major Cities of Australia | 164 (85%) | 30 (15%) |
|  | Regional and Remote Australia | 153 (83%) | 32 (17%) |
| Do you have children under 5? | No | 199 (84%) | 38 (16%) |
|  | Yes | 118 (83%) | 24 (17%) |
| What education level have you completed? | Did Not Complete High School | 115 (83%) | 24 (17%) |
|  | Completed High School | 131 (87%) | 19 (13%) |
|  | Completed Tertiary Education | 71 (79%) | 19 (21%) |

**Table S8: Associations Between Characteristics and Owning a “Mobile Phone”**

| *Characteristic* | *Crude* *(n=379)*  *OR* *(95%CI)* | *p* | *Adjusted* *(n=379)*  *OR* *(95%CI)* | *p* |
| --- | --- | --- | --- | --- |
| Age (+5 year increment) | 1.247 (1.036, 1.500) | 0.0194 | 1.222 (1.006, 1.484) | 0.0436 |
| ARIA+ (Regional and Remote Australia vs. Major Cities of Australia) | 1.143 (0.663, 1.971) | 0.6297 | 1.204 (0.689, 2.103) | 0.5155 |
| Do you have any children under 5? (Yes vs. No) | 1.065 (0.609, 1.864) | 0.8251 | 1.194 (0.672, 2.119) | 0.5452 |
| What education level have you completed? (Overall comparison*) |  | 0.2209* |  | 0.4558* |
| Competed Tertiary Education vs. Completed High School | 1.845 (0.918, 3.710) | 0.0856 | 1.590 (0.766, 3.301) | 0.2135 |
| Did Not Complete High School vs. Completed High School | 1.439 (0.750, 2.762) | 0.2739 | 1.299 (0.668, 2.526) | 0.4401 |

**Table S9: Participant Characteristics for Selecting “telehealth”**

| *Characteristic* *Class/Statistic* *Unchecked* | | | *Checked* |
| --- | --- | --- | --- |
| *(n=227)* | | | *(n=152)* |
| Age | <21 | 29 (67%) | 14 (33%) |
|  | 21-34 | 124 (63%) | 72 (37%) |
|  | >34 | 74 (53%) | 66 (47%) |
| ARIA+ | Major Cities of Australia | 108 (56%) | 86 (44%) |
|  | Regional and Remote Australia | 119 (64%) | 66 (36%) |
| Do you have children under 5? | No | 146 (62%) | 91 (38%) |
|  | Yes | 81 (57%) | 61 (43%) |
| What education level have you completed? | Did Not Complete High School | 87 (63%) | 52 (37%) |
|  | Completed High School | 87 (58%) | 63 (42%) |
|  | Completed Tertiary Education | 53 (59%) | 37 (41%) |

**Table S10: Associations Between Characteristics and Selecting “telehealth”**

| *Characteristic* | *Crude* *(n=379)*  *OR* *(95%CI)* | *p* | *Adjusted* *(n=379)*  *OR* *(95%CI)* | *p* |
| --- | --- | --- | --- | --- |
| Age (+5 year increment) | 1.198 (1.044, 1.374) | 0.0102 | 1.232 (1.065, 1.425) | 0.0049 |
| ARIA+ (Regional and Remote Australia vs. Major Cities of Australia) | 0.697 (0.461, 1.053) | 0.0862 | 0.695 (0.455, 1.062) | 0.0926 |
| Do you have any children under 5? (Yes vs. No) | 1.208 (0.792, 1.844) | 0.3807 | 1.286 (0.834, 1.984) | 0.2551 |
| What education level have you completed? (Overall comparison*) |  | 0.7111* |  | 0.4648* |
| Competed Tertiary Education vs. Completed High School | 0.964 (0.567, 1.639) | 0.8924 | 0.754 (0.430, 1.324) | 0.3261 |
| Did Not Complete High School vs. Completed High School | 0.825 (0.515, 1.324) | 0.4259 | 0.759 (0.466, 1.236) | 0.2682 |

**Table S11: Participant Characteristics for Selecting “Video Conferencing***”*

| *Characteristic* *Class/Statistic* *Unchecked* | | | *Checked* |
| --- | --- | --- | --- |
| *(n=279)* | | | *(n=100)* |
| Age | <21 | 33 (77%) | 10 (23%) |
|  | 21-34 | 142 (72%) | 54 (28%) |
|  | >34 | 104 (74%) | 36 (26%) |
| ARIA+ | Major Cities of Australia | 141 (73%) | 53 (27%) |
|  | Regional and Remote Australia | 138 (75%) | 47 (25%) |
| Do you have children under 5? | No | 176 (74%) | 61 (26%) |
|  | Yes | 103 (73%) | 39 (27%) |
| What education level have you completed? | Did Not Complete High School | 114 (82%) | 25 (18%) |
|  | Completed High School | 104 (69%) | 46 (31%) |
|  | Completed Tertiary Education | 61 (68%) | 29 (32%) |

**Table S12: Associations Between Characteristics and Selecting “Video Conferencing”**

| *Characteristic* | *Crude* *(n=379)*  *OR* *(95%CI)* | *p* | *Adjusted* *(n=379)*  *OR* *(95%CI)* | *p* |
| --- | --- | --- | --- | --- |
| Age (+5 year increment) | 0.999 (0.860, 1.160) | 0.9899 | 1.003 (0.856, 1.176) | 0.9677 |
| ARIA+ (Regional and Remote Australia vs. Major Cities of Australia) | 0.906 (0.573, 1.432) | 0.6726 | 0.972 (0.608, 1.552) | 0.9040 |
| Do you have any children under 5? (Yes vs. No) | 1.092 (0.683, 1.747) | 0.7121 | 1.102 (0.683, 1.778) | 0.6901 |
| What education level have you completed? (Overall comparison*) |  | 0.0196* |  | 0.0210* |
| Competed Tertiary Education vs. Completed High School | 1.075 (0.613, 1.886) | 0.8013 | 1.077 (0.597, 1.945) | 0.8051 |
| Did Not Complete High School vs. Completed High School | 0.496 (0.285, 0.863) | 0.0132 | 0.497 (0.284, 0.872) | 0.0147 |

**Table S13: Participant Characteristics for Selecting “A Text Messaging Service”**

| *Characteristic* *Class/Statistic* *Unchecked* | | | *Checked* |
| --- | --- | --- | --- |
| *(n=168)* | | | *(n=211)* |
| Age | <21 | 18 (42%) | 25 (58%) |
|  | 21-34 | 85 (43%) | 111 (57%) |
|  | >34 | 65 (46%) | 75 (54%) |
| ARIA+ | Major Cities of Australia | 89 (46%) | 105 (54%) |
|  | Regional and Remote Australia | 79 (43%) | 106 (57%) |
| Do you have children under 5? | No | 103 (43%) | 134 (57%) |
|  | Yes | 65 (46%) | 77 (54%) |
| What education level have you completed? | Did Not Complete High School | 64 (46%) | 75 (54%) |
|  | Completed High School | 66 (44%) | 84 (56%) |
|  | Completed Tertiary Education | 38 (42%) | 52 (58%) |

**Table S14: Associations Between Characteristics and Selecting “A Text Messaging Service”**

| *Characteristic* | *Crude* *(n=379)*  *OR* *(95%CI)* | *p* | *Adjusted* *(n=379)*  *OR* *(95%CI)* | *p* |
| --- | --- | --- | --- | --- |
| Age (+5 year increment) | 0.996 (0.872, 1.137) | 0.9534 | 0.990 (0.862, 1.136) | 0.8810 |
| ARIA+ (Regional and Remote Australia vs. Major Cities of Australia) | 1.137 (0.758, 1.706) | 0.5342 | 1.162 (0.770, 1.753) | 0.4738 |
| Do you have any children under 5? (Yes vs. No) | 0.910 (0.599, 1.383) | 0.6603 | 0.910 (0.597, 1.388) | 0.6626 |
| What education level have you completed? (Overall comparison*) |  | 0.8463* |  | 0.7991* |
| Competed Tertiary Education vs. Completed High School | 1.075 (0.634, 1.823) | 0.7879 | 1.102 (0.634, 1.914) | 0.7313 |
| Did Not Complete High School vs. Completed High School | 0.921 (0.579, 1.464) | 0.7272 | 0.916 (0.572, 1.465) | 0.7129 |

**Table S15: Participant Characteristics for Selecting “Social Media”**

| *Characteristic* *Class/Statistic* *Unchecked* | | | *Checked* |
| --- | --- | --- | --- |
| *(n=184)* | | | *(n=195)* |
| Age | <21 | 21 (49%) | 22 (51%) |
|  | 21-34 | 94 (48%) | 102 (52%) |
|  | >34 | 69 (49%) | 71 (51%) |
| ARIA+ | Major Cities of Australia | 94 (48%) | 100 (52%) |
|  | Regional and Remote Australia | 90 (49%) | 95 (51%) |
| Do you have children under 5? | No | 113 (48%) | 124 (52%) |
|  | Yes | 71 (50%) | 71 (50%) |
| What education level have you completed? | Did Not Complete High School | 75 (54%) | 64 (46%) |
|  | Completed High School | 67 (45%) | 83 (55%) |
|  | Completed Tertiary Education | 42 (47%) | 48 (53%) |

**Table S16: Associations Between Characteristics and Selecting “Social Media”**

| *Characteristic* | *Crude* *(n=379)*  *OR* *(95%CI)* | *p* | *Adjusted* *(n=379)*  *OR* *(95%CI)* | *p* |
| --- | --- | --- | --- | --- |
| Age (+5 year increment) | 0.979 (0.858, 1.117) | 0.7489 | 0.984 (0.857, 1.129) | 0.8153 |
| ARIA+ (Regional and Remote Australia vs. Major Cities of Australia) | 0.992 (0.663, 1.485) | 0.9697 | 1.020 (0.677, 1.536) | 0.9260 |
| Do you have any children under 5? (Yes vs. No) | 0.911 (0.601, 1.382) | 0.6617 | 0.901 (0.592, 1.372) | 0.6278 |
| What education level have you completed? (Overall comparison*) |  | 0.2656* |  | 0.2716* |
| Competed Tertiary Education vs. Completed High School | 0.923 (0.546, 1.559) | 0.7632 | 0.931 (0.538, 1.612) | 0.7993 |
| Did Not Complete High School vs. Completed High School | 0.689 (0.433, 1.095) | 0.1150 | 0.691 (0.432, 1.105) | 0.1224 |

**Table S17: Participant Characteristics for Selecting “Health Apps”**

| *Characteristic* *Class/Statistic* *Unchecked* | | | *Checked* |
| --- | --- | --- | --- |
| *(n=195)* | | | *(n=184)* |
| Age | <21 | 26 (60%) | 17 (40%) |
|  | 21-34 | 91 (46%) | 105 (54%) |
|  | >34 | 78 (56%) | 62 (44%) |
| ARIA+ | Major Cities of Australia | 103 (53%) | 91 (47%) |
|  | Regional and Remote Australia | 92 (50%) | 93 (50%) |
| Do you have children under 5? | No | 118 (50%) | 119 (50%) |
|  | Yes | 77 (54%) | 65 (46%) |
| What education level have you completed? | Did Not Complete High School | 70 (50%) | 69 (50%) |
|  | Completed High School | 82 (55%) | 68 (45%) |
|  | Completed Tertiary Education | 43 (48%) | 47 (52%) |

**Table S18: Associations Between Characteristics and Selecting “Health Apps”**

| *Characteristic* | *Crude* *(n=379)*  *OR* *(95%CI)* | *p* | *Adjusted* *(n=379)*  *OR* *(95%CI)* | *p* |
| --- | --- | --- | --- | --- |
| Age (+5 year increment) | 0.993 (0.870, 1.132) | 0.9125 | 0.967 (0.843, 1.110) | 0.6366 |
| ARIA+ (Regional and Remote Australia vs. Major Cities of Australia) | 1.144 (0.764, 1.712) | 0.5128 | 1.172 (0.778, 1.765) | 0.4477 |
| Do you have any children under 5? (Yes vs. No) | 0.837 (0.552, 1.270) | 0.4031 | 0.842 (0.553, 1.283) | 0.4245 |
| What education level have you completed? (Overall comparison*) |  | 0.5567* |  | 0.5061* |
| Competed Tertiary Education vs. Completed High School | 1.318 (0.781, 2.226) | 0.3015 | 1.375 (0.794, 2.381) | 0.2555 |
| Did Not Complete High School vs. Completed High School | 1.189 (0.749, 1.888) | 0.4639 | 1.192 (0.746, 1.906) | 0.4624 |

**Table S19: Participant Characteristics for Selecting “Health Tracker”**

| *Characteristic* *Class/Statistic* *Unchecked* | | | *Checked* |
| --- | --- | --- | --- |
| *(n=215)* | | | *(n=164)* |
| Age | <21 | 24 (56%) | 19 (44%) |
|  | 21-34 | 104 (53%) | 92 (47%) |
|  | >34 | 87 (62%) | 53 (38%) |
| ARIA+ | Major Cities of Australia | 113 (58%) | 81 (42%) |
|  | Regional and Remote Australia | 102 (55%) | 83 (45%) |
| Do you have children under 5? | No | 132 (56%) | 105 (44%) |
|  | Yes | 83 (58%) | 59 (42%) |
| What education level have you completed? | Did Not Complete High School | 73 (53%) | 66 (47%) |
|  | Completed High School | 92 (61%) | 58 (39%) |
|  | Completed Tertiary Education | 50 (56%) | 40 (44%) |

**Table S20: Associations Between Characteristics and Selecting “Health Tracker”**

| *Characteristic* | *Crude* *(n=379)*  *OR* *(95%CI)* | *p* | *Adjusted* *(n=379)*  *OR* *(95%CI)* | *p* |
| --- | --- | --- | --- | --- |
| Age (+5 year increment) | 1.013 (0.887, 1.157) | 0.8475 | 0.991 (0.863, 1.139) | 0.9020 |
| ARIA+ (Regional and Remote Australia vs. Major Cities of Australia) | 1.135 (0.756, 1.705) | 0.5411 | 1.135 (0.751, 1.715) | 0.5489 |
| Do you have any children under 5? (Yes vs. No) | 0.894 (0.587, 1.361) | 0.6004 | 0.904 (0.591, 1.382) | 0.6400 |
| What education level have you completed? (Overall comparison*) |  | 0.3098* |  | 0.3264* |
| Competed Tertiary Education vs. Completed High School | 1.269 (0.747, 2.156) | 0.3783 | 1.292 (0.742, 2.251) | 0.3648 |
| Did Not Complete High School vs. Completed High School | 1.434 (0.898, 2.289) | 0.1309 | 1.427 (0.888, 2.292) | 0.1414 |

**Table S21: Participant Characteristics for Selecting “Cultural Engagement”**

| *Characteristic* *Class/Statistic* *Unchecked* | | | *Checked* |
| --- | --- | --- | --- |
| *(n=174)* | | | *(n=205)* |
| Age | <21 | 26 (60%) | 17 (40%) |
|  | 21-34 | 91 (46%) | 105 (54%) |
|  | >34 | 57 (41%) | 83 (59%) |
| ARIA+ | Major Cities of Australia | 69 (36%) | 125 (64%) |
|  | Regional and Remote Australia | 105 (57%) | 80 (43%) |
| Do you have children under 5? | No | 102 (43%) | 135 (57%) |
|  | Yes | 72 (51%) | 70 (49%) |
| What education level have you completed? | Did Not Complete High School | 74 (53%) | 65 (47%) |
|  | Completed High School | 66 (44%) | 84 (56%) |
|  | Completed Tertiary Education | 34 (38%) | 56 (62%) |

**Table S22: Associations Between Characteristics and Selecting “Cultural Engagement”**

| *Characteristic* | *Crude* *(n=379)*  *OR* *(95%CI)* | *p* | *Adjusted* *(n=379)*  *OR* *(95%CI)* | *p* |
| --- | --- | --- | --- | --- |
| Age (+5 year increment) | 1.092 (0.956, 1.247) | 0.1942 | 1.081 (0.938, 1.247) | 0.2812 |
| ARIA+ (Regional and Remote Australia vs. Major Cities of Australia) | 0.421 (0.278, 0.636) | <.0001 | 0.437 (0.287, 0.666) | 0.0001 |
| Do you have any children under 5? (Yes vs. No) | 0.735 (0.484, 1.115) | 0.1476 | 0.752 (0.488, 1.159) | 0.1968 |
| What education level have you completed? (Overall comparison*) |  | 0.0615* |  | 0.1761* |
| Competed Tertiary Education vs. Completed High School | 1.294 (0.758, 2.208) | 0.3443 | 1.028 (0.580, 1.822) | 0.9236 |
| Did Not Complete High School vs. Completed High School | 0.690 (0.434, 1.097) | 0.1169 | 0.670 (0.414, 1.085) | 0.1034 |

**Table S23: Participant Characteristics for Selecting “Healthy Eating”**

| *Characteristic* *Class/Statistic* *Unchecked* | | | *Checked* |
| --- | --- | --- | --- |
| *(n=169)* | | | *(n=210)* |
| Age | <21 | 16 (37%) | 27 (63%) |
|  | 21-34 | 92 (47%) | 104 (53%) |
|  | >34 | 61 (44%) | 79 (56%) |
| ARIA+ | Major Cities of Australia | 85 (44%) | 109 (56%) |
|  | Regional and Remote Australia | 84 (45%) | 101 (55%) |
| Do you have children under 5? | No | 100 (42%) | 137 (58%) |
|  | Yes | 69 (49%) | 73 (51%) |
| What education level have you completed? | Did Not Complete High School | 63 (45%) | 76 (55%) |
|  | Completed High School | 64 (43%) | 86 (57%) |
|  | Completed Tertiary Education | 42 (47%) | 48 (53%) |

**Table S24: Associations Between Characteristics and Selecting “Healthy Eating”**

| *Characteristic* | *Crude* *(n=379)*  *OR* *(95%CI)* | *p* | *Adjusted* *(n=379)*  *OR* *(95%CI)* | *p* |
| --- | --- | --- | --- | --- |
| Age (+5 year increment) | 1.031 (0.903, 1.177) | 0.6495 | 1.039 (0.905, 1.194) | 0.5856 |
| ARIA+ (Regional and Remote Australia vs. Major Cities of Australia) | 0.938 (0.625, 1.406) | 0.7555 | 0.929 (0.615, 1.401) | 0.7238 |
| Do you have any children under 5? (Yes vs. No) | 0.772 (0.508, 1.173) | 0.2256 | 0.770 (0.505, 1.174) | 0.2243 |
| What education level have you completed? (Overall comparison*) |  | 0.8139* |  | 0.6782* |
| Competed Tertiary Education vs. Completed High School | 0.850 (0.503, 1.438) | 0.5459 | 0.785 (0.452, 1.364) | 0.3909 |
| Did Not Complete High School vs. Completed High School | 0.898 (0.564, 1.429) | 0.6494 | 0.874 (0.545, 1.401) | 0.5763 |

**Table S25: Participant Characteristics for Selecting “Exercise”**

| *Characteristic* *Class/Statistic* *Unchecked* | | | *Checked* |
| --- | --- | --- | --- |
| *(n=217)* | | | *(n=162)* |
| Age | <21 | 31 (72%) | 12 (28%) |
|  | 21-34 | 107 (55%) | 89 (45%) |
|  | >34 | 79 (56%) | 61 (44%) |
| ARIA+ | Major Cities of Australia | 106 (55%) | 88 (45%) |
|  | Regional and Remote Australia | 111 (60%) | 74 (40%) |
| Do you have children under 5? | No | 135 (57%) | 102 (43%) |
|  | Yes | 82 (58%) | 60 (42%) |
| What education level have you completed? | Did Not Complete High School | 83 (60%) | 56 (40%) |
|  | Completed High School | 83 (55%) | 67 (45%) |
|  | Completed Tertiary Education | 51 (57%) | 39 (43%) |

**Table S26: Associations Between Characteristics and Selecting “Exercise”**

| *Characteristic* | *Crude* *(n=379)*  *OR* *(95%CI)* | *p* | *Adjusted* *(n=379)*  *OR* *(95%CI)* | *p* |
| --- | --- | --- | --- | --- |
| Age (+5 year increment) | 1.095 (0.958, 1.252) | 0.1836 | 1.109 (0.964, 1.276) | 0.1468 |
| ARIA+ (Regional and Remote Australia vs. Major Cities of Australia) | 0.803 (0.534, 1.208) | 0.2919 | 0.807 (0.534, 1.222) | 0.3117 |
| Do you have any children under 5? (Yes vs. No) | 0.968 (0.636, 1.475) | 0.8813 | 0.992 (0.648, 1.518) | 0.9703 |
| What education level have you completed? (Overall comparison*) |  | 0.7477* |  | 0.6217* |
| Competed Tertiary Education vs. Completed High School | 0.947 (0.559, 1.604) | 0.8404 | 0.822 (0.473, 1.429) | 0.4868 |
| Did Not Complete High School vs. Completed High School | 0.836 (0.524, 1.334) | 0.4521 | 0.800 (0.497, 1.288) | 0.3587 |

**Table S27: Relationship Between Confidence to Discuss Health Topics and likelihood of selecting that topic for mHealth**

*Interested* *in* *Using* *a* *Mobile* *Phone* *for* *Health* *Topic*

*Confident* *to* *Discuss*

| *Health* *Topic* | *Health* *Topic* *with* *a* *Health* *Professional?* | *Not* *Selected* *in* *Top* *3* | *Selected* *in* *Top* *3* | *p* |
| --- | --- | --- | --- | --- |
| Eating and diet | No | 25 (16%) | 26 (13%) | 0.4464 |
|  | Yes | 136 (84%) | 178 (87%) |  |
|  | Not Relevant | 8 | 6 |  |
| Reducing alcohol | No | 36 (19%) | 11 (48%) | 0.0020 |
|  | Yes | 150 (81%) | 12 (52%) |  |
|  | Not Relevant | 170 | 0 |  |
| Exercise | No | 32 (16%) | 19 (12%) | 0.2853 |
|  | Yes | 168 (84%) | 139 (88%) |  |
|  | Not Relevant | 17 | 4 |  |
| Family violence | No | 73 (38%) | 6 (25%) | 0.1992 |
|  | Yes | 117 (62%) | 18 (75%) |  |
|  | Not Relevant | 161 | 4 |  |
| Quitting smoking | No | 31 (16%) | 18 (15%) | 0.8942 |
|  | Yes | 165 (84%) | 100 (85%) |  |
|  | Not Relevant | 61 | 4 |  |
| Cannabis or other drug use | No | 50 (37%) | 18 (41%) | 0.6458 |
|  | Yes | 85 (63%) | 26 (59%) |  |
|  | Not Relevant | 196 | 4 |  |
| Mental health | No | 26 (14%) | 24 (16%) | 0.6522 |
|  | Yes | 158 (86%) | 127 (84%) |  |
|  | Not Relevant | 40 | 4 |  |
| Womens health | No | 29 (9.6%) | 7 (11%) | 0.6494 |
|  | Yes | 274 (90%) | 54 (89%) |  |
|  | Not Relevant | 15 | 0 |  |
| Childs health | No | 11 (4.0%) | 1 (2.2%) | 0.5530 |
|  | Yes | 261 (96%) | 44 (98%) |  |
|  | Not Relevant | 62 | 0 |  |
